# Varicella zoster immune globulin (VARIZIG) administration up to 10 days after varicella exposure in pregnant women, immunocompromised participants, and infants: Varicella outcomes and safety results from a large, open-label, expanded-access program

**DOI:** 10.1371/journal.pone.0217749

**Published:** 2019-07-03

**Authors:** Myron J. Levin, Jennifer M. Duchon, Geeta K. Swamy, Anne A. Gershon

**Affiliations:** 1 Department of Infectious Diseases, University of Colorado Anschutz Medical Campus, Aurora, Colorado, United States of America; 2 Department of Neonatal-Perinatal Medicine, Tufts Floating Hospital for Children, Boston, Massachusetts, United States of America; 3 Department of Obstetrics and Gynecology, Duke University, Durham, North Carolina, United States of America; 4 Department of Pediatrics, Columbia University College of Physicians and Surgeons, New York, New York, United States of America; Public Health England, UNITED KINGDOM

## Abstract

**Introduction:**

Despite vaccination, there were more than 100,000 annual cases of varicella in the United States in 2013–2014. Individuals at highest risk of developing severe or complicated varicella include immunocompromised people, preterm infants, and pregnant women. Varicella zoster immune globulin (human) (VARIZIG) is recommended by the CDC for postexposure prophylaxis to prevent or attenuate varicella-zoster virus infection in high-risk individuals. Contemporary information on administration of VARIZIG is limited.

**Methods:**

This open-label, expanded-access program provided VARIZIG to physician-identified, high-risk participants exposed to varicella. Participants included immunocompromised children/adults, infants (preterm, newborns whose mothers had varicella onset within 5 days before or 2 days after delivery, and those aged <1 year), and pregnant women. VARIZIG (125 IU/10 kg [up to 625 IU]) was administered intramuscularly, ideally within 96 hours, but up to 10 days, postexposure. Incidence of varicella rash and severity (>100 pox, pneumonia, or encephalitis) were assessed up to 42 days after administration.

**Results:**

The varicella outcome population (n = 507) included 263 immunocompromised participants (32 adults, 231 children), 137 pregnant women, 105 infants, and 2 healthy adults with no history of varicella. Varicella incidence was 4.5% in immunocompromised participants, 7.3% in pregnant women, and 11.5% in infants. The incidence of varicella was similar when comparing VARIZIG administration ≤ 96 hours vs > 96 hours (up to 10 days) postexposure in the entire population (6.2% vs. 9.4%, respectively), and also in each subgroup. Of 34 participants with varicella, 5 developed > 100 pox and 1 developed pneumonia and encephalitis. There were no product-related deaths and only 1 serious adverse event (serum sickness) considered probably related to VARIZIG.

**Conclusion:**

Postexposure administration of VARIZIG was associated with low rates of varicella in high-risk participants, regardless of when administered within 10 days postexposure. VARIZIG was well-tolerated and safe in high-risk participants.

## Introduction

Although the incidence of varicella has dropped dramatically in the United States because of varicella vaccination, as of 2014 there remained more than 100,000 annual cases of varicella [1,2]. In addition, there are an estimated 1 million cases of shingles each year in the United States [3]. Exposure of high-risk individuals to varicella-zoster virus (VZV) can result in severe or complicated varicella including pneumonitis, encephalitis, and hepatitis [4]. People at highest risk of developing severe or complicated varicella include non-immune adults, immunocompromised patients [4–8], and preterm infants [4,9,10]. Pregnant women are more likely to develop varicella pneumonia than non-pregnant adults [11], particularly if they have ≥ 100 pox and are current smokers [12], although the rate of varicella pneumonia may be lower than previously reported [13]. Children born to pregnant women who develop varicella are at risk of the sequelae of congenital varicella syndrome, depending on when exposure occurs during the pregnancy; however, it is unknown if passive immunization will alter the risk for congenital varicella syndrome [4,7,9,12,14–16]. Newborn infants whose mothers had onset of varicella within 5 days before delivery or within 48 hours after delivery are at especially high risk from severe varicella, presumably because they do not receive protective transplacental VZV antibodies before birth [7,17,18].

The long incubation period of varicella provides an opportunity to interrupt infection and attenuate clinical varicella and its complications. Since the 1960s, clinicians have used non-specific immune serum globulin [19], zoster immune globulin (ZIG) prepared from the plasma of individuals recovering from shingles [20–22] and a previous formulation of varicella zoster immune globulin (known as VZIG) prepared from individuals with high titers of varicella immunoglobulin [8,23] to prevent or reduce the severity of varicella [24]. However, data have suggested that administration of VZIG beyond 3 days after exposure was associated with declining efficacy, leaving a narrow treatment window [25]. Furthermore, in 2004, VZIG was discontinued, with little supply remaining by 2006 [26].

In 2006, a new post-exposure prophylactic product was approved by the US Food and Drug Administration (FDA) to replace discontinued VZIG [26]. Varicella zoster immune globulin (human) (VARIZIG, Saol Therapeutics, Roswell, Georgia) is recommended for post-exposure prophylaxis to prevent or attenuate varicella-zoster virus infection in high-risk individuals [27]. VARIZIG is recommended to be administered as soon as possible following exposure, ideally within 96 hours; however, the Centers for Disease Control and Prevention (CDC) recommends that it can be administered as late as 10 days after exposure [28]. VARIZIG, which is prepared from plasma donated by healthy donors with high titers of VZV antibodies because of recent VZV infection, is now widely available [27]. In the only randomized, active-controlled clinical trial comparison of VARIZIG (pre-FDA approval) and discontinued VZIG in 60 pregnant women without evidence of varicella immunity, the incidence of varicella was not significantly different between the 2 hyperimmune globulin preparations (VARIZIG: n = 5 of 17 [29%]; VZIG: n = 8 of 19 [42%]) and each treatment exhibited similar safety outcomes [29]. Given the limited clinical information with VARIZIG and the paucity of contemporary data on passive immunization to prevent or attenuate varicella in high-risk populations, this study was designed to assess varicella incidence and severity in high-risk participants after administration of VARIZIG in a pragmatic, real-world clinical setting.

## Methods

### Participants

Physician-identified, high-risk participants exposed to VZV were eligible for the study. The protocol did not specify what constituted an exposure to varicella or herpes zoster, which was left to the judgment of the investigator. Exposure was classified as household, hospital, in utero, workplace, daycare, direct contact outside the household (included physical contact with an infected individual), or other. More than 1 type of exposure could have occurred (eg, household and direct contact). The duration of VZV exposure and timing of administration postexposure were defined based on the description provided to the site investigator by the participant, a family member, or medical personnel. High-risk participants included immunocompromised children and adults, pregnant women, and infants (including preterm infants, newborns whose mother had varicella < 5 days before or within 2 days after delivery, and infants aged < 1 year). Healthy adults without evidence of varicella immunity who were exposed to varicella or herpes zoster were also eligible.

Participants were excluded from the study if they had known immunity to VZV, hypersensitivity to blood or blood products including human immunoglobulin preparations, hypersensitivity to any component of VARIZIG, a history of selective immunoglobulin A (IgA) deficiency, evidence of current VZV infection (chicken pox or shingles) at study entry, or evidence of severe thrombocytopenia.

### Study design and treatment

This was an open-label, expanded-access program in a real-world setting (NCT00338442) that took place at 285 clinical study sites across the United States (in 49 states, the District of Columbia, and Puerto Rico) and was completed between March 2006 and April 2013. There were 4 study visits, including a baseline visit (determination of eligibility, VZV exposure history, administration of VARIZIG, and adverse event [AE] monitoring in the immediate post-VARIZIG administration period), an observational visit between days 1 to 4 (to collect data on safety and clinical outcomes), a second observational visit between days 7 to 20, and a visit between days 28 to 42 (or on study withdrawal) to complete the assessment of potential varicella infection and completion of AE and safety data. VARIZIG (125 IU/10 kg [up to 625 IU]) was administered intramuscularly, ideally within 96 hours post-exposure, but as late as 10 days, post-exposure. The minimum dose was 125 IU (1 vial) for participants ≤ 10 kg body weight; all participants weighing > 40 kg received 625 IU (5 vials). In the initial protocol, a reduced dose (62.5 IU, one-half vial) was allowed for infants weighing < 5 kg and provisions were provided for intravenous dosing. Based on feedback from the FDA and CDC, and recommendations made by the Advisory Committee on Immunization Practices, the minimum dose was revised to 125 IU in subsequent protocol versions; intravenous dosing was also removed from the protocol.

This study was conducted in accord with the Good Clinical Practice Guideline as defined by the International Conference on Harmonisation, the Declaration of Helsinki, and all applicable federal and local regulation and institutional review board guidelines, as appropriate. The protocol and amendments, the informed consent form, and study-related materials were reviewed and approved by a central independent ethics committee, Western Institutional Review Board (Puyallup, WA), before study initiation and throughout the conduct of the study. All patients (or their guardians) provided written informed consent.

### Assessments

#### Clinical outcomes

Assessments were measured at each visit, up to 42 days after administration of VARIZIG. At each visit, participants were evaluated for the presence of varicella lesions. Complications of varicella were recorded, including pneumonia, encephalitis, or mortality. Cases with > 100 pox were defined as “severe”. The primary endpoint was the incidence of varicella, as defined by the investigator based on occurrence of typical rash. Secondary endpoints included severity of varicella defined by pox count and/or evidence of organ involvement. The use of concomitant antiviral medications for VZV, including acyclovir and valacyclovir were recorded.

#### Safety assessment

Patient reports of AEs were recorded through 42 days after VARIZIG administration. AEs were coded by the MedDRA (version 16.0) coding system. All AEs were assessed for seriousness, severity, and causality by the investigator. An AE was considered severe if it resulted in inability to work or undertake usual daily activities. AEs were considered serious if they resulted in death, were life-threatening, required hospitalization, resulted in persistent or significant disability/incapacity, or if it was a congenital anomaly/birth defect.

### Study populations and statistical analysis

**Fig 1:**
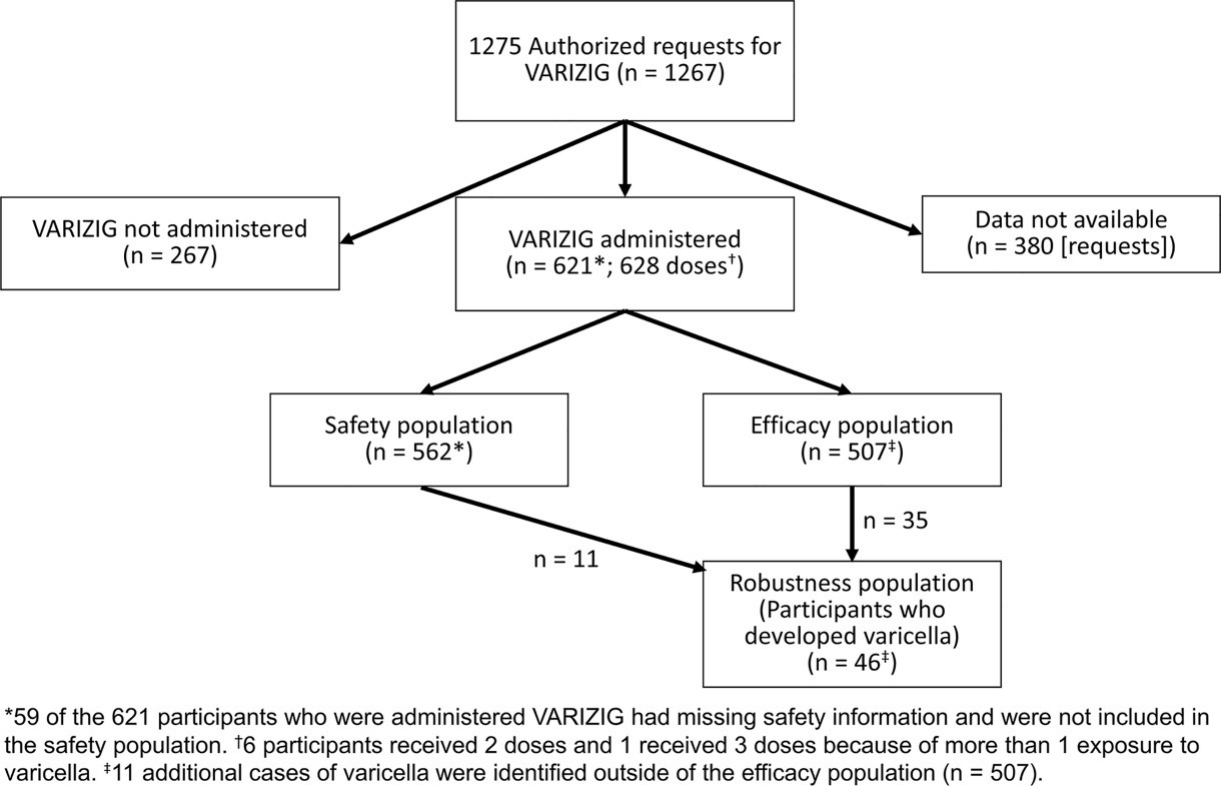
Participant disposition.

The safety population included all participants who received VARIZIG and had complete case report forms (Fig 1). The varicella outcome population was defined as all participants who had definitive varicella outcome data. Participants who withdrew early or were lost to follow-up with ≤ 21 days of follow-up information were excluded from the varicella outcome analysis. To ensure that all participants who developed varicella were captured, those with incomplete varicella outcome data (ie, the pre-defined clinical review of varicella was not complete) who had sufficient data pertinent to varicella noted elsewhere were included in a population to assess the robustness of the data (robustness population). The population of healthy adults with no history of varicella or vaccination was not analyzed as a separate subgroup because this population did not meet the minimum sample size of 30 participants. Data were analyzed using descriptive statistics. The incidence of varicella after administration of VARIZIG within 96 hours and > 96 hours through 10 days after exposure was compared using a Fisher’s exact test at a 5% level of significance. *P* values were considered significant if *P* < .05. All analyses were performed using SAS (Cary, NC) version 9.4.

### Description of protocol deviations

Three protocol versions were implemented in this study (version 1, 3, and 4; version 2 was approved but never implemented because its timing overlapped with version 3). Initially, both intramuscular and intravenous dosing of VARIZIG were planned, consistent with the approved Canadian label. However, intravenous dosing was removed in version 2. In addition, the original protocol provided provisions for a reduced dose of VARIZIG (62.5 IU, one-half vial) for infants weighing less than 5 kg; however, based on feedback from the CDC, the minimum dose was revised to 125 IU in version 3. There were 11 children weighing under 5 kg who received less than 125 IU VARIZIG; some received 62.5 IU and some received a lower dose because of extreme low weight (eg, a 1 kg preterm infant received 50 IU). Even at these lower doses, the dose per kilogram of 125 IU/10 kg was exceeded in these children.

Inclusion of healthy adults lacking VZV immunity was added in version 3. This study was originally designed to enroll and treat participants exposed to VZV within the first 96 hours of exposure; however, in version 4, the study population was expanded to enroll high-risk participants exposed to VZV ideally within the first 96 hours but up to 10 days post exposure. The analysis to compare varicella incidence based on timing of administration was added in version 4. The robustness analysis was not specified in the protocol but was added to the statistical analysis plan. The protocol specified that laboratory data were to be collected at each study visit; however, the prespecified summary of laboratory data over time was not executed because of missing data and lack of consistency between study sites.

## Results

### Participants

Of 1275 authorized requests, data were returned for 915 participants. Of these, VARIZIG was administered to 621 participants and was not administered to 267 participants because they did not meet eligibility requirements; 7 participants received > 1 dose and data were unavailable for 380 participants (Fig 1). The safety population included 562 participants; 59 participants received VARIZIG but were excluded from the safety population because of incomplete safety information. The varicella outcome population included 507 participants. Most participants in this population were also in the safety population; however, 23 participants in the varicella outcome population were excluded from the safety population because of incomplete information. The disposition of participants who received VARIZIG by risk type is shown in Table 1. Of note, 7 immunocompromised participants received more than 1 dose of VARIZIG (6 participants received 2 doses and 1 participant received 3 doses) because of more than 1 exposure to VZV at least 1 month apart; each of these exposures/doses was independently considered. Preterm infants ranged in age from 0 days to 6 months; in the varicella outcome population, the majority of preterm infants were ≤ 1 month (n = 42), nearly all were ≤ 2 months (n = 51), 3 were 3 months, 1 was 5 months, and 1 was 6 months. There were 5 healthy adults included in the study who had no history of varicella who received VARIZIG; of these, 4 participants were included in the safety population and 2 were included in the varicella outcome population, but they were excluded from the risk group analyses because of the small group size.

**Table 1:** Distribution of participants who received VARIZIG.

	Participants, n (%)		
High-risk Participant Type	All Participants*	Safety Population	Varicella Outcome Population)
	(n = 621)	(n = 562)	(n = 507)
Healthy adults with no history of varicella	5 (0.8)	4 (0.7)	2 (0.4)
Pregnant women	166 (27)	147 (26)	137 (27)
Immunocompromised participants^†^	299 (48)	272 (48)	263 (52)
Immunocompromised adults (aged > 18 years)	40 (6)	37 (7)	32 (6)
Immunocompromised children^†^ (aged ≤ 18 years)	259 (42)	235 (42)	231 (46)
Infants	152 (24)	139 (25)	105 (20)
Full-term newborns, aged 0–27 days	52 (8)	48 (9)	38 (7)
Preterm infants, aged 0 days– 6 months	86 (14)	79 (14)	56 (11)
Infants, aged 28 days– 1 year	14 (2)	12 (2)	11 (2)

*59 of the 621 participants who were administered VARIZIG had missing safety information and were not included in the safety population

^†^7 participants received >1 dose (6 received 2 doses and 1 received 3 doses) because these participants had >1 exposure to varicella (> 1 month apart). Each exposure/dose was considered independent

Demographics and baseline characteristics at the time of administration of VARIZIG are shown in Table 2. Exposure of pregnant women to VZV most commonly occurred in the household; of immunocompromised individuals in a household or hospital setting; and in infants within a hospital setting. Most participants did not receive acyclovir; however, more immunocompromised participants received acyclovir than pregnant women or infants (28% vs. 10% and 14%, respectively). Classification of a participant as “immunocompromised” was most frequently because of chemotherapy to treat malignancy or the use of immunosuppressive agents to support solid organ or stem cell transplantation.

**Table 2:** Baseline demographics (safety population).

			Immunocompromised Participants			
Demographic		Pregnant Women	All	Adults	Pediatric	Infants
		(n = 147)	(n = 272)	(n = 37)	(n = 235)	(n = 139)
Age, years (months for infants)	Mean (SD)	29.1 (6.32)	11.9 (14.78)	43.9 (17.01)	6.9 (4.69)	1.2 (1.96)
	Median	30	7	40	6	0
	Range	16–43	0–71	18–71	0–18	0–12
Sex, n (%)	Female	147 (100)	132 (49)	17 (46)	115 (49)	63 (45)
	Male	**—**	140 (51)	20 (54)	120 (51)	76 (55)
Race, n (%)	White	79 (54)	175 (64)	25 (68)	150 (64)	62 (45)
	Hispanic or Latino	32 (22)	45 (17)	4 (11)	41 (17)	35 (25)
	Black or African American	11 (8)	33 (12)	5 (14)	28 (12)	22 (16)
	Asian	8 (5)	5 (2)	1 (3)	4 (2)	10 (7)
	Not reported/declined/Other	17 (11)	14 (5)	2 (5)	12 (5)	10 (7)
Weight, kg	Mean (SD)	79.8 (22.00)	33.0 (23.95)	73.5 (14.58)	26.6 (18.19)	2.8 (1.59)
	Median	75	23	73	20	3
	Range	40–146	1–126	50–126	1–95	1–10
Acyclovir use, n (%)	Yes	14 (10)	76 (28)	11 (30)	65 (28)	20 (14)
	No	133 (90)	196 (72)	26 (70)	170 (72)	119 (86)
Exposure type, n (%)*	Non-household direct contact with lesions	28 (19)	22 (8)	4 (11)	18 (8)	27 (19)
	Household	69 (47)	85 (31)	17 (46)	68 (29)	25 (18)
	At work	26 (18)	**—**	**—**	**—**	**—**
	At daycare	4 (3)	15 (6)	**—**	15 (6)	**—**
	Hospital	14 (10)	95 (35)	11 (30)	84 (36)	84 (60)
	Other^†^	33 (22)	74 (27)	8 (22)	66 (28)	38 (27)

The group of healthy adults without evidence of immunity (n = 4) was comprised of 3 women and 1 man; 2 were white, 1 Hispanic, and 1 not reported. Participants were, on average, 34.1 (11.51) years old, weighed 67.3 (6.75) kg, did not use acyclovir (100%), and were exposed in the home (n = 2) or hospital (n = 1) (1 exposure was not reported)

*Participants could have more than 1 type of exposure

^†^Other exposures included: exposure to infected mother around infant delivery, exposure via classroom/church/playgroup, exposure while being held by a person with chickenpox/shingles, and exposure in a clinic waiting area or by an infected healthcare provider

*SD* standard deviation

### Varicella incidence in VARIZIG recipients

#### Participant type and timing

**Fig 2:**
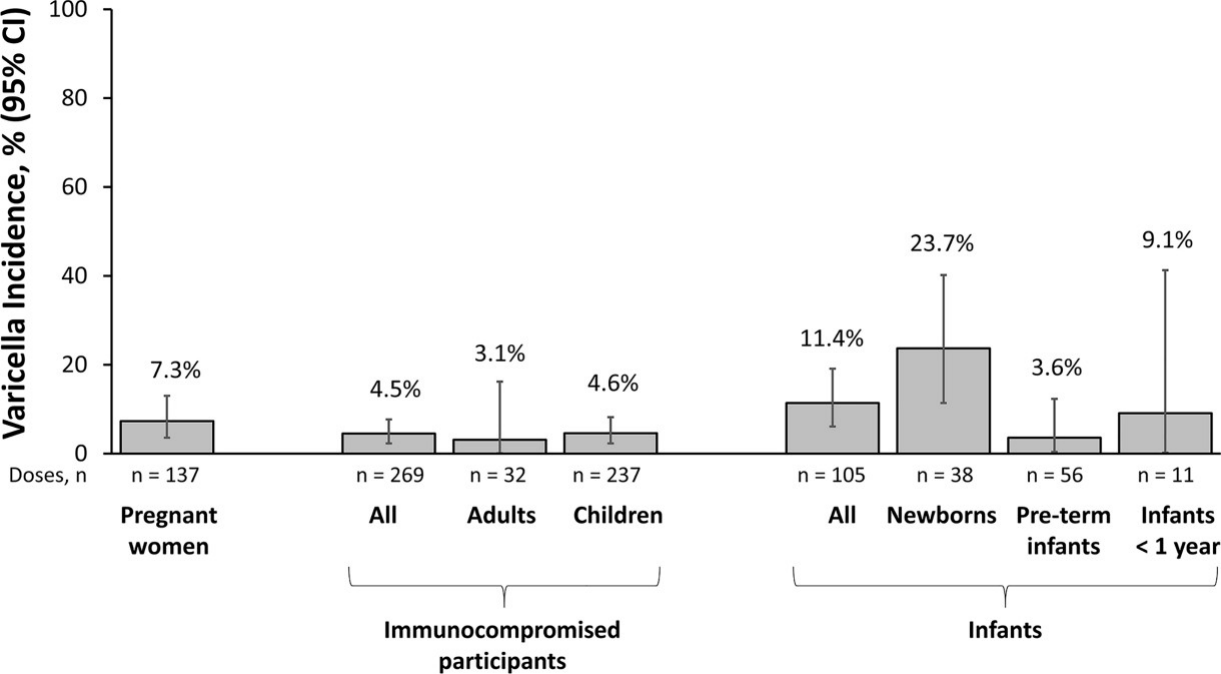
Incidence of varicella by high-risk participant group. *CI* confidence interval.

The incidence of varicella was 4.5% (95% confidence interval [CI]: 2.3%– 7.7%) in immunocompromised participants, 7.3% (95% CI: 3.6%– 13.0%) in pregnant women, and 11.4% (95% CI: 6.1%– 19.1%) in infants; the highest incidence of varicella was in newborns born to mothers who experienced chickenpox < 5 days before or within 2 days of birth (23.7%; 95% CI: 11.4%– 40.2%) (Fig 2). Of the 2 healthy adults with no history of varicella who received VARIZIG and had varicella outcome data, 1 who received VARIZIG within 96 hours of exposure developed varicella that was mild and without any reported complications of varicella.

**Fig 3:**
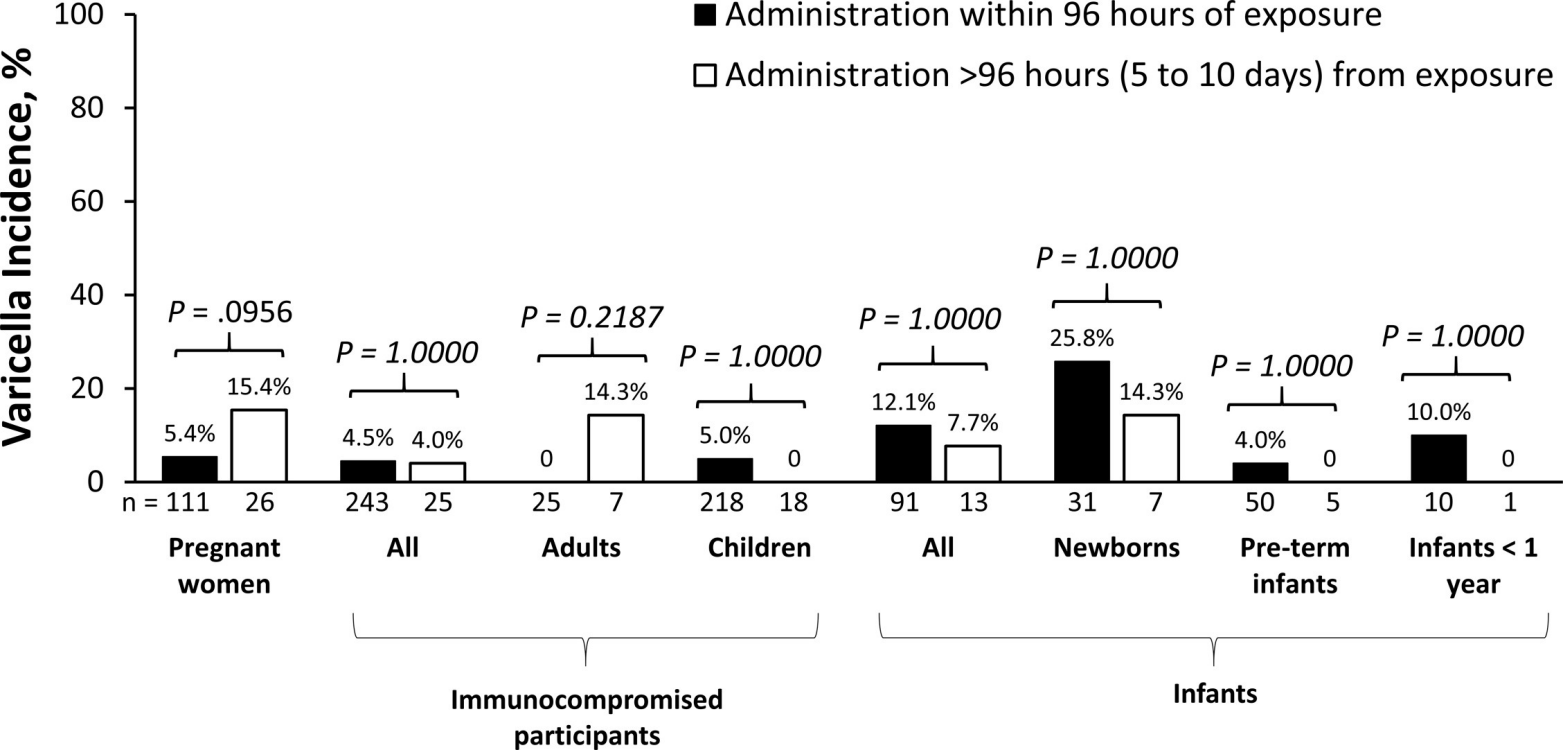
Incidence of varicella by timing of administration. *P* values were determined using a 2-sided Fisher’s exact test. *CI* confidence interval.

The incidence of varicella was not significantly different, regardless of the timing of administration (within 96 hours of exposure vs. > 96 hours [up to 10 days] after exposure) for the entire population (6.3% vs. 9.4%, respectively) and also in each high-risk group (Fig 3). Among participants who developed varicella, all but 2 were administered VARIZIG within 6 days of exposure, and 38 of 46 (83%) received VARIZIG within the first 96 hours after exposure.

#### Exposure type

There were 46 cases of varicella (35 cases in the varicella outcome analysis population and 11 additional cases in the robustness population). Of participants who developed varicella, most reported household exposure (n = 26, 56.5%) or direct contact with lesions (n = 14, 30.4%); however, patients could report more than 1 type of exposure (eg, household and direct contact). Fourteen participants (30.4%) cited “other reasons” for exposure, including exposure to infected mother around delivery, exposure at classroom/church/playgroup, and exposure in a clinic waiting area or by an infected healthcare provider. No participants cited daycare as an exposure location. Five participants who developed varicella reported only exposure to herpes zoster (household exposure, n = 2; hospital setting, n = 2; exposure to mother with herpes zoster at birth, n = 1).

#### Evidence of modified disease

Five of 35 participants with varicella in the varicella outcome population developed > 100 pox (2 immunocompromised participants and 3 infants). Each received VARIZIG within 96 hours of exposure to varicella. None of the 10 evaluable participants with varicella in the robustness population had > 100 pox. No pregnant women developed > 100 pox or had any complications. One newborn developed disseminated varicella with pneumonia and encephalitis after intrauterine exposure to VZV; this patient received acyclovir treatment at birth and received VARIZIG 6 days after birth. When this patient began to exhibit evidence of clinical varicella, acyclovir was administered again. No participants died because of varicella.

#### Concomitant acyclovir use

**Fig 4:**
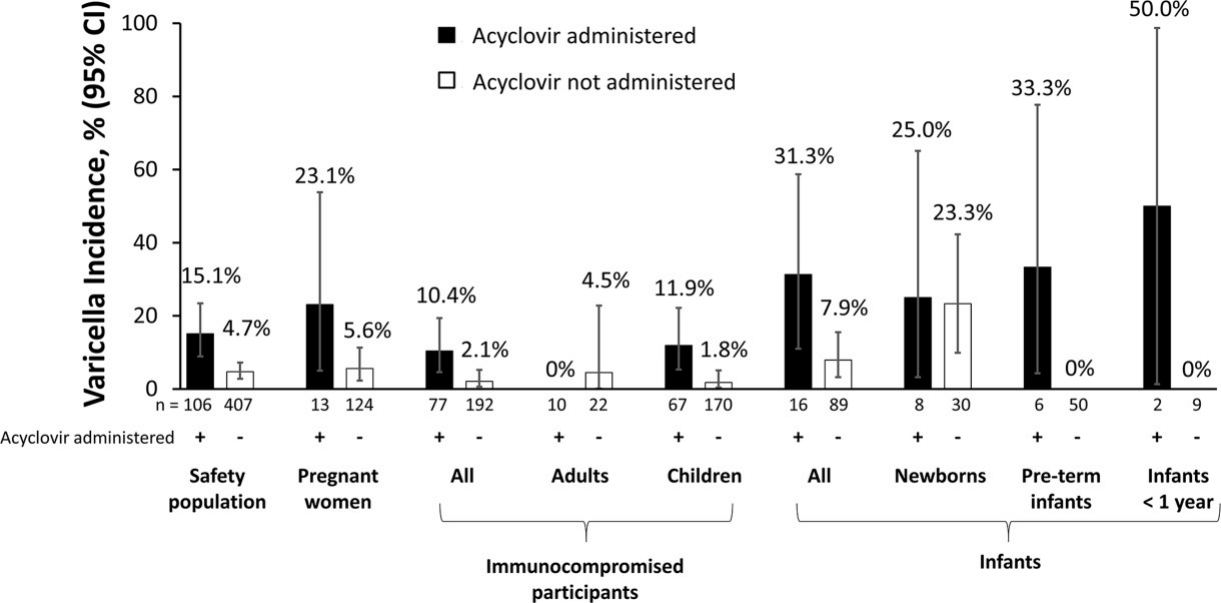
Incidence of varicella by absence or presence of concomitant antiviral therapy. *CI* confidence interval.

Of the 106 participants who received prophylactic acyclovir or valacyclovir concomitantly with VARIZIG, 16 participants (15.1%) developed varicella; of the remaining 407 participants who did not receive concomitant antiviral therapy, 19 participants (4.7%) developed varicella (Fig 4). In the individual high-risk participant groups, there was a trend for lower incidence of varicella in participants who did not receive acyclovir compared with participants who did receive acyclovir; but the number of participants in some subgroups was limited. Of the 46 participants who developed varicella, 19 (41.3%) received concomitant prophylactic acyclovir or valacyclovir.

### Safety

A total of 159 participants (28%) experienced 598 AEs; 81 AEs were considered to be related to VARIZIG (Table 3). Immunocompromised participants experienced the majority (76%) of AEs reported in the study. AEs were reported in 10% of pregnant women and in 13% of infants. The most common AE (≥ 3% of participants) was pyrexia; all reports of pyrexia occurred in immunocompromised participants and only 1 report was considered related to VARIZIG. A total of 62 participants (11%) experienced 142 serious AEs, 6 of which were considered possibly related to VARIZIG (convulsion, headache, nausea, serum sickness, vomiting). Only 1 serious AE (serum sickness) diagnosed in a child was considered probably related to VARIZIG. Serum sickness occurred 5 days following administration of VARIZIG and was not considered related to the child’s underlying leukemia. Of the 4 healthy adults with no history of varicella, 2 participants reported 4 AEs, of which only 1 AE of nasopharyngitis was considered possibly related to treatment. There were 8 deaths during the study (6 immunocompromised participants [5 children and 1 adult], 2 preterm infants). None of the deaths were considered to be related to VARIZIG and they did not occur in participants with varicella.

**Table 3:** Safety summary.

	Participants, n (%)			
Parameter	Pregnant Women	Immunocompromised Participants	Infants	Safety Population
	(n = 147)	(n = 272)	(n = 139)	(n = 562)
**Adverse events (AEs)**				
Participants, n (%)	23 (16)	102 (38)	32 (23)	159 (28)
Total AEs, n	58	456	80	598
**Most common AEs (≥3% of participants)**				
Pyrexia	**—**	23 (9)	**—**	23 (4)
AEs related to VARIZIG*				
Participants, n (%)	13 (9)	17 (6)	4 (3)	35 (6)
Total AEs, n	17	56	4	78
**Participants with related AEs (in ≥2 participants)**^**†**^				
Injection site pain	4 (3)	4 (2)	**—**	8 (1)
Headache	2 (1)	3 (1)	**—**	5 (0.9)
Nausea	1 (0.7)	3 (1)	**—**	4 (0.7)
Fatigue	2 (1)	1 (0.4)	**—**	3 (0.5)
Diarrhea	**—**	2 (0.7)	**—**	2 (0.4)
Vomiting	**—**	2 (0.7)	**—**	2 (0.4)
**Serious AEs**				
Participants, n (%)	4 (3)	44 (16)	13 (9)	62 (11)
Total serious AEs, n	4	109	26	142
**Participants with related serious AEs**	**—**	2 (0.7)	2 (1.4)	4 (0.7)
Convulsion	**—**	**—**	1 (0.7)	1 (0.2)
Headache	**—**	1 (0.4)	**—**	1 (0.2)
Nausea	**—**	1 (0.4)	**—**	1 (0.2)
Serum sickness	**—**	1 (0.4)	**—**	1 (0.2)
Varicella	**—**	**—**	1 (0.7)	1 (0.2)
Vomiting	**—**	1 (0.4)	**——**	1 (0.2)
**Deaths (not related to treatment)**^**‡**^	**—**	5 (2)	2 (1)	7 (1)

In adults without evidence of immunity (n = 4), 2 participants experienced 4 adverse events (AEs) (neutropenia, nausea, vomiting, and nasopharyngitis); 1 of these AEs (nasopharyngitis) was considered related to VARIZIG

*The investigator determined whether an AE was considered related to treatment

^†^Infants experienced 1 each of the following related AEs: convulsion, flushing, irritability, and varicella

^‡^One additional participant died from causes unrelated to treatment or varicella, but from causes related to the subject’s underlying condition. This participant was not included in the safety population because the participant died before completing the study

## Discussion

Since 2002 [29], there have been no publications reporting clinical experience with passive immunization to prevent varicella, and the most recent systematic review on the topic was published in 2011 [24]. Because of revised guidelines, confusion between discontinued VZIG and subsequent availability of VARIZIG, and no recent publications, there has been uncertainty about proper management of susceptible individuals exposed to varicella or herpes zoster. In addition, with the increased prevalence of vaccine refusers, the need for passive immunization for high-risk individuals has increased [30].

In this expanded-access program in which the determination of risk, exposure, and clinical outcome was made by each local investigator, the incidence and severity of varicella was low in high-risk participants after administration of VARIZIG. Although there was no comparator for this study, these results can be evaluated in the context of clinical outcomes after similar exposures in the era before passive immunization was available. In this study, rates of varicella in high-risk populations after VARIZIG prophylaxis were 4.5%, 7.3%, and 11.4% in immunocompromised participants, pregnant women, and infants, respectively. In a retrospective study that covered a 24-year period, the overall rate of varicella in 5777 pediatric patients with cancer was 5% (including 45 patients who received passive immunization) [31], similar to the rate in the current study. The rate of visceral dissemination reported for immunocompromised children ranges from 21% to 48% and rates of mortality range from 7% to 36% [5,31–34]. By comparison, in the current study no immunocompromised child experienced visceral dissemination or varicella-related death.

An Italian retrospective study reported that in pregnant women exposed to VZV, 72% of those who did not receive post-exposure prophylaxis developed varicella compared with 42% of those who received VZIG (discontinued 2006) [35]. In the current study, the rate of varicella in pregnant women was much lower, at 7.3%. Rates of varicella-associated complications such as pneumonia reportedly occur in 2.5% to 20% of varicella cases in pregnant women [13,36]; no pregnant women developed pneumonia in the current study. The rate of varicella in the current study (7.3%) was lower than previously reported (29%) in a randomized controlled trial comparing VARIZIG and VZIG (discontinued 2006) [29]. This difference in varicella incidence could be related to the stringency of the controlled trial in determining if a participant’s exposure required prophylaxis, or it could be because a larger percentage of patients in the current study received VARIZIG within 96 hours of exposure than in the controlled trial (81% vs 65%, respectively); however, our results indicate that the timing of administration does not affect the incidence of varicella.

The peripartum subgroup exhibits the clearest connection between maternal varicella and infant exposure. In the newborn subgroup (n = 38), the incidence of varicella was 23.7%. Previous studies of VZIG (discontinued 2006) treatment in neonates following perinatal exposure reported rates of mild disease ranging from 32% to 67% and severe disease ranging from 0% to 11% of neonates [37]. In the current study, only 1 infant developed disseminated varicella, which was manifest as pneumonia. The reported mortality rate of varicella in newborns exposed around birth ranges from 14% to 31% [9,38]; there were no varicella-related deaths in newborns in the current study.

Although we did not assess the incidence of varicella in the overall population by timing of VARIZIG administration, in this study, varicella rates were similar for participants administered VARIZIG within 96 hours of exposure compared with those administered VARIZIG after 96 hours and up through 10 days after exposure. These data support the CDC recommendations to administer VARIZIG as late as 10 days after exposure to varicella [28]. Complications related to varicella were rare, and treatment with VARIZIG was well-tolerated and appeared to be safe in high-risk participants.

Between 10% and 30% of participants (depending on the subgroup) received prophylactic acyclovir or valacyclovir treatment. Although there was a trend for lower incidence of varicella in those who did not receive anti-VZV nucleoside analogues compared with those who did (4.7% vs. 15.1%, respectively, in the overall population), their use in this study was based on the decision of each investigator and assignment was not randomized or controlled. Consequently, these data are likely to be confounded by baseline patient factors and cannot be used to make recommendations about concomitant use of nucleoside analogues with VARIZIG. Although there are no official guidelines for the use of antiviral prophylaxis against varicella, prevention of varicella has been reported when antiviral therapy was started between 6 and 10 days after exposure and continued for 7 days compared with initiating antiviral therapy between 0 to 3 days after exposure [39]. In the current expanded-access program, less than 30% of the participants who received antiviral prophylaxis followed this course of antiviral treatment. Most participants received antiviral prophylaxis immediately after exposure (often before administration of VARIZIG) and treatment was often shorter than 7 days. The antiviral drugs are widely used for treatment of varicella infection and for post-exposure prophylaxis treatment (oftentimes as adjunctive therapy to VZIG) for immunocompromised patients, with variable success [6,24,40,41]. Few publications reported on acyclovir use as a solitary post-exposure prophylactic agent [24]. Bate *et al*. were unable to evaluate the efficacy of a different formulation of VZIG made in Europe vs. acyclovir as post-exposure prophylaxis in immunocompromised children [40].

This was an open-label, single-arm study, without a comparator intervention arm. Expanded-access program evaluation is encouraged by the FDA to assess investigational new drugs for serious or life-threatening diseases that lack therapeutic alternatives [42]. Unlike controlled trials, expanded-access programs provide information about the use of drugs in a real-word clinical practice setting. With respect to VARIZIG, the nature of this program sometimes resulted in insufficient detail about VZV exposure and the risk status of persons receiving post-exposure prophylaxis. However, a thorough review of exposure in all participants concluded that the exposures described in the case report forms were consistent with the CDC’s definition of exposure (ie, > 5 minutes of face-to-face contact with an infectious person while indoors, but more typically > 1 hour of exposure). Incomplete case report forms (10% missing) also imposed some limits on the conclusions that can be drawn from these data. Notwithstanding the limitations inherent in this open-label, single-arm study in a real-world clinical setting, these results are consistent with current recommendations and guidance to the practicing clinician faced with a vulnerable patient exposed to VZV.

## Conclusions

This large study conducted in 3 different high-risk populations exposed to varicella, demonstrates that VARIZIG can be safely administered up to 10 days in patients after their exposure to varicella and likely reduces the incidence of varicella. These data are consistent with the current CDC recommendations for the use of hyperimmune globulin to prevent varicella in high-risk populations.

## Supporting information

S1 Checklist TREND checklist. (DOCX)

S1 Protocol Study protocol. (PDF)
