# Supplementary material for: Varicella zoster immune globulin (VARIZIG) administration up to 10 days after varicella exposure in pregnant women, immunocompromised participants, and infants: Varicella outcomes and safety results from a large, open-label, expanded-access program
Source: PLoS One. 2019 Jul 3;14(7):e0217749. doi: 10.1371/journal.pone.0217749 (PMC6608934; doi:10.1371/journal.pone.0217749)
Supplement: S1 Protocol — (PDF) [file pone.0217749.s002.pdf]

CANGENE

Expanded Access Protocol

Safety and Efficacy of Varicella Zoster Immune Globulin (Human) (VariZIG™) in Patients At-Risk of Varicella Infection

Version 1.0

November 22, 2005

Sponsor:
Cangene Corporation
26 Henlow Bay
Winnipeg, Manitoba
R3Y 1G4

Distributor:
[redacted]

Protocol Number VZ-009
Version 1.0
November 22, 2005

Investigator: T.B.D.

Institution/CRO: T.B.D.

Trial Sponsor:

Cangene Corporation
26 Henlow Bay
Winnipeg, MB R3Y 1G4

Clinical Study Scientist:

Name: [redacted]

Tel: [redacted]

Fax: [redacted]

E-mail: [redacted]

Signatory Page

Protocol VZ-009, version 1.0 : **Safety and Efficacy of Varicella Zoster Immune Globulin (Human) (VariZIG™) in Patients At-Risk of Varicella Infection**

Clinical Site:

Name: ______________________

Address: ______________________

____

Telephone: ______________________

My signature below verifies that I have read and approved this protocol. I am aware of my responsibilities as an Investigator under the GCP guidelines of ICH, the Declaration of Helsinki, local regulations (as applicable) and the study protocol, and I agree to conduct the study according to these regulations.

Site Principal Investigator:

Principal Investigator Name (print)	Title (print)
Principal Investigator Signature	Date (yyyy/mmm/dd)

Sponsor Signatory:

[redacted]

2005-Nov-22
Date (yyyy/mmm/dd)

Cangene Corporation
26 Henlow Bay
Winnipeg, MB
Canada R3Y 1G4

VZ-009 Protocol Synopsis

**Title**	Safety and Efficacy of Varicella Zoster Immune Globulin (Human) (VariZIG™) in Patients At-Risk of Varicella Infection
**Sponsor**	Cangene Corporation<br>26 Henlow Bay<br>Winnipeg, Manitoba, Canada, R3Y 1G4
**Site & Investigator**	T.B.D.
**Study Start**	T.B.D.
**Enrolment Complete**	T.B.D.
**Follow-up Complete**	T.B.D.
**Objectives**	• To outline the handling and use of VariZIG™ which is to be distributed by [redacted] under an expanded access protocol.<br>• To collect safety and efficacy data for VariZIG™.
**Patient Population**	Any of the following at-risk patients exposed to varicella:<br>• Immune compromised pediatric patients.<br>• Immune compromised adult patients.<br>• Neonates (less than 1 year of age).<br>• Pre-term infants.<br>• Pregnant women.<br>• Newborns whose mothers had varicella zoster virus (VZV) infection shortly before (less than 5 days) delivery.<br>• Newborns whose mothers had VZV infection shortly after (less than 2 days) delivery.
**Sample Size**	All eligible consented patients will be enrolled in this study. There is no upper limit for the number of patients enrolled. For evaluation of the secondary endpoint, a minimum of 16 immune compromised patients enrolled into this study will be compared to untreated historical control data for the development of pneumonia in at-risk patients exposed to

	VZV (chicken pox) infection. This will allow 80% power to detect a 25% difference between patients treated with VariZIG™ and historical control values.
**Test Product**	VariZIG™
**Dosage**	For patients greater than 5 kg:<br>125 IU (one vial of VariZig™) / 10 kg body weight IM, to a maximum dose of 625 IU.<br><br>For patients less than 5 kg:<br>62.5 IU (one-half vial of VariZIG™) should be administered IM. This is approximately 0.6 mL per dose, which will minimize any adverse effects, especially in pre-term infants.
**Protocol Design**	• This is an open-label expanded access protocol to assess the safety and efficacy of VariZIG™ in the prevention or reduction of complications resulting from varicella zoster virus (VZV) infections in at-risk patients exposed to individuals with infectious VZV infections.<br>• VariZIG™ is distributed by [redacted] for release on an individual patient basis.<br>• After determination of the need for VariZIG™ by the investigator, the investigator will contact [redacted] or Cangene Corporation for shipment of VariZIG™. After Cangene Corporation (or designate) reviews patient eligibility and approves the request, the investigator's and the patient's information will be entered into a database and VariZIG™, the protocol, investigator brochure (IB), and informed consent form (ICF) will be shipped from [redacted] to the investigator.<br>• [redacted] will notify Cangene Corporation (or designate) about the shipment of VariZIG™ and the requesting investigator's information.<br>• Prior to VariZIG™ administration to the patient, the patient (or legal representative) must sign an ICF that has been approved by a central institutional review board (IRB).<br>• [redacted] or Cangene Corporation will contact the

	investigator within 7 days of VariZIG™ shipment for follow-up and to discuss data collection.<br>• [redacted] or Cangene Corporation will contact the investigator at least 3 times by phone in order to collect data.
**Inclusion Criteria**	• Signed informed consent.<br>• Cangene Corporation VariZIG™ release requirement.<br>• Any of the following at-risk patients exposed to varicella:<br>• Immunocompromised pediatric or adult patients.<br>• Neonates (<1 year of age) and pre-term infants.<br>• Pregnant women.<br>• Newborns whose mothers had VZV infection shortly before (<5 days) or after (<2 days) delivery.
**Exclusion Criteria**	• Hypersensitivity to blood or blood products, including IV or IM human immunoglobulin preparations.<br>• Selective immunoglobulin A (IgA) deficiency.<br>• Evidence of VZV infection.<br>• Evidence of Zoster infection.
**Assessments**	**Visit 1 (Baseline/Day 0)**<br>• Informed consent.<br>• Basic medical history (especially information related to varicella exposure including type, time since exposure, and duration of exposure).<br>• Collection of the following pre-treatment lab data if available:<br>• Hematology assessments: hemoglobin, hematocrit, WBC, RBC indices, and platelet count.<br>• Blood Chemistry: total bilirubin, AST, ALT, alkaline phosphatase, lactate dehydrogenase, creatinine, BUN.<br>• Administration of VariZIG™.<br><br>**Visit 2 (Day 1 to 4)**<br>• Adverse event and concomitant medication information.<br>• Evaluation of varicella lesion(s), if applicable: type,

	number, size, location on the body and percent of body area affected.<br>• Collection of the following data if available:<br>• Hematology assessments: hemoglobin, hematocrit, WBC, RBC indices and platelet count.<br>• Blood Chemistry: total bilirubin, AST, ALT, alkaline phosphatase, lactate dehydrogenase, creatinine, BUN.<br><br>**Visit 3 (Day 7 to Day 20 (Approximate day of varicella rash development as applicable)):**<br>• Adverse event and concomitant medication information.<br>• Review of varicella lesion(s), if applicable: type, number, size, location on the body and percent of body area affected.<br>• Collection of the following data if available:<br>• Hematology assessments: hemoglobin, hematocrit, WBC, RBC indices and platelet count.<br>• Blood Chemistry: total bilirubin, AST, ALT, alkaline phosphatase, lactate dehydrogenase, creatinine, BUN.<br><br>**Visit 4 (Day 28 to 42 (closeout)):**<br>• Patients should return for a closeout visit upon resolution of the chicken pox rash, or on Day 42, whichever occurs first.<br>• Adverse event and concomitant medication information.<br>• Review of varicella lesion(s), if applicable: type, number, size, location on the body and percent of body area affected.<br>• Clinical review of varicella infection severity, including development of pneumonia, encephalitis and >100 pox on chest/back.<br>• Collection of the following data if available:<br>• Hematology assessments: hemoglobin, hematocrit,

	WBC, RBC indices and platelet count.<br>• Blood Chemistry: total bilirubin, AST, ALT, alkaline phosphatase, lactate dehydrogenase, creatinine, BUN.
**Safety Parameters**	Adverse events related to VariZIG™ will be collected throughout the study.
**Concomitant Medications**	A log of concomitant medications including transfusions, herbal preparations and non-prescription medications must be kept throughout the study.
**Outcomes**	• Incidence of adverse events.<br>• Incidence of mortality.<br>• Percent of patients with pox count >100.<br>• Incidence of pneumonia and encephalitis.<br>• Overall incidence of varicella infection.<br>• Clinical observations and laboratory assessments.

Schedule of Events

	VISIT 1	VISIT 2	VISIT 3	VISIT 4
	**Baseline (Day 0)**	**Day 1 to Day 4**	**Day 7 to Day 20 (Approximate day of varicella rash development as applicable)[1]**	**Day 28 to Day 42 (closeout)**
Admission Criteria	X			
Informed Consent	X			
Medical History	X			
History of Varicella Exposure	X			
Hematology	X	X	X	X
Blood Chemistry	X	X	X	X
VariZIG™ Dosing[2]	X			
Adverse Events	X[3]	X	X	X
Concomitant Medications	X[3]	X	X	X
Evaluation of varicella lesion(s) (as appropriate)		X	X	X
Clinical review of Varicella infection				X

[1]If appropriate. [2]Re-dosing of VariZIG™ may occur on Days other than Day 0, if clinically justified. [3]Post dosing.

FIGURE 1: Schematic Diagram of VariZIG™ Release and Data Collection

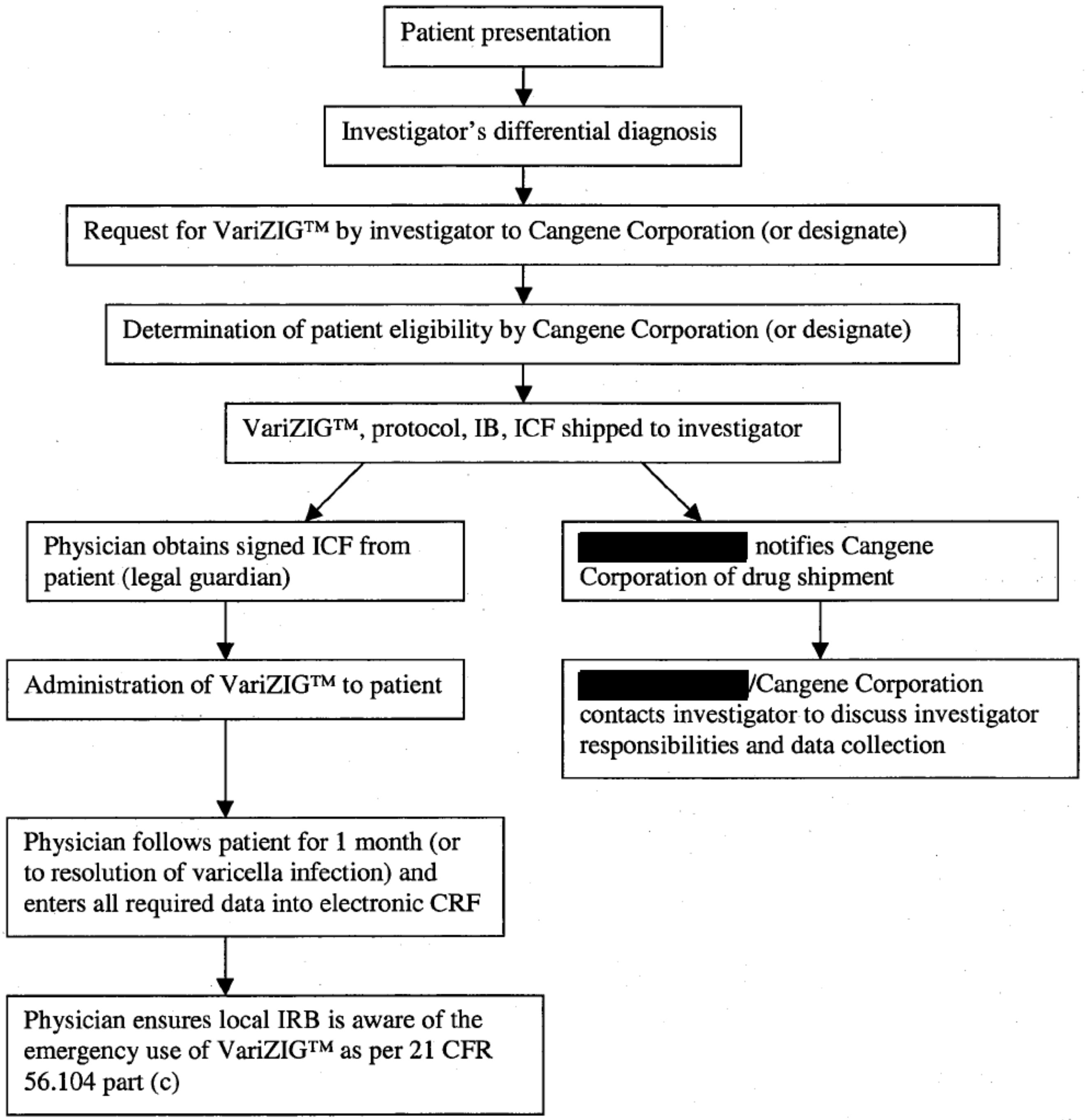

Table of Contents

1 OBJECTIVES 15
2 BACKGROUND RATIONALE 15
2.1 Study Drug 15
2.2 Clinical Study Rationale 15
2.3 Hypothesis 18
3 STUDY DESIGN 18
3.1 Study Design 18
3.2 Anticipated Centers 18
3.3 Sample Size 19
4 ELIGIBILITY REQUIREMENTS 19
4.1 Inclusion Criteria 19
4.2 Exclusion Criteria 19
4.3 Excluded Concomitant Medications 19
5 STUDY PROCEDURES 19
5.1 Randomization 19
5.2 Baseline and Baseline Assessment 20
5.3 Drug Administration 20
5.4 Study Assessment 20
5.5 Laboratory Assessments 22
6 SAFETY ASSESSMENTS 22
6.1 Adverse Event/Serious Adverse Event Definition 22
6.1.1 Definitions 22
6.1.2 Assessment of Severity (Intensity) 23
6.1.3 Assessment of Causality (WHO Definitions) 23
6.1.4 Description of Known Adverse Event Profile for VariZIG™ 24
6.2 Adverse Event Reporting 24
6.3 Reporting of SAEs 24
7 STUDY MEDICATION 25
7.1 Medication Shipment 25
7.2 Packaging and Formulation 25
7.3 Labeling 25
7.4 Storage Conditions 26
7.5 Preparation 26
7.6 Drug Accountability 27
8 STATISTICAL ISSUES IN STUDY DESIGN AND EFFICACY ASSESSMENT 27
8.1 Sample Size 27
8.2 Study Endpoints 27
8.2.1 Primary Endpoint 27
8.2.2 Secondary Endpoints 28
8.3 Planned Method of Analyses 28

9 REGULATORY AND ETHICAL ISSUES 29
9.1 Informed Consent 29
9.2 Institutional Review Board (IRB) 30
9.3 Study Documentation 30
9.4 Patient Confidentiality 31
10 ADMINISTRATIVE AND LEGAL REQUIREMENTS 31
10.1 Sponsorship 31
10.2 Protocol Amendments 31
10.3 Study Termination 32
10.4 Source Documentation and Storage 32
10.5 Electronic Data Capture 32
10.6 Publication 32
11 REFERENCES 33

List of Tables

Table 1: Incidence and Severity of VZV Infections in At-Risk Individuals 17
Table 2: Incidence and Severity of VZV Infection in Immunocompromised Children 17
Table 3: Reconstitution of VariZIG™ 26
Table 4: Historical Rate of Development of Pneumonia in At-Risk Patients Untreated and Treated with VZIG 28

List of Abbreviations and Definition of Terms

CRF	Case report form
ICF	Informed consent form
IRB	Institutional review board
VariZIG™	Cangene Corporation's varicella zoster immune globulin
VZIG	Varicella zoster immune globulin
VZV	Varicella zoster virus

1 OBJECTIVES

- To outline the handling and use of VariZIG™ which is to be distributed by [redacted] under an expanded access protocol.
- To collect safety and efficacy data for VariZIG™.

2 BACKGROUND RATIONALE

2.1 Study Drug

Trade Name: VariZIG™

Established Name: Varicella Zoster Immune Globulin (Human) Injection

Abbreviated Name: VZIG

Laboratory Code: NP-001

VariZIG™ is a sterile freeze-dried gamma globulin (IgG) fraction of human plasma that containing antibodies to varicella zoster virus (anti-VZV). Varicella zoster virus (VZV) is the causative agent of chickenpox. VariZIG™ is manufactured from plasma collected from healthy screened donors with high titres of anti-VZV, which is purified by an anion-exchange column chromatography method[1,2].

VariZIG™ is supplied as a lyophilized cake in a 6 mL serum glass vial sealed with a butyl rubber stopper and an aluminum seal with a plastic flip-off cap. Each package also contains 8.5 mL of sterile diluent for reconstitution of VariZIG™. Potency of VariZIG™ is expressed in international units (IU) by comparison to the World Health Organization (WHO) international anti-varicella zoster immune globulin reference preparation. Each vial contains greater than 125 IU/mL of anti-VZV. The final product formulation includes addition of sodium chloride to yield 0.04 M, glycine to yield 0.1 M, and polysorbate 80 to yield 0.01%. The accompanying sterile diluent contains 0.8% sodium chloride and 10 mM sodium phosphate. The reconstituted product contains no preservative.

2.2 Clinical Study Rationale

Varicella zoster virus (VZV) infection typically causes a benign highly contagious disease that is characterized by discrete pruritic vesicles that present from head to trunk. Prior to vesicle eruption, systemic features including fever, chills, myalgia and arthralgia are present

for 2 to 3 days. From exposure, the incubation period is usually 14 to 15 days until vesicle eruption, with 95% of patients developing a rash between 11 to 20 days after exposure[3]. During the first 48 hours of vesicle eruption, the patient is considered contagious. In non-immune individuals, varicella zoster virus is one of the most contagious viral agents. Secondary infections in at-risk individuals following household exposure produce infection rates of >85% while tertiary contacts yield infections in approximately 70% of individuals[4]. This indicates that a non-immune person would have at least a 70% chance of developing chicken pox if exposed to a contagious person[5]. The most likely source of infection is close contact including household exposure[4,6] although workplace and daycare exposures[7] may also be important in VZV transmission. Similar rates of infection have been reported in newborns whose mothers developed chicken pox less than 5 days prior to delivery[8].

In North America and Europe, the majority of VZV cases (~95%) occur in children less than 15 years of age. Prior to the licensure of VZV vaccine (Varivax®, Merck), less than 10% of adults in North America were not immune to VZV infection. This number is anticipated to decrease over the next few decades in individuals born and raised in North America.

In the immune competent host (especially children), VZV disease is typically benign. However, VZV disease can produce significant morbidity and mortality in certain patient populations. For example, immigrants to North America from tropical/sub-tropical regions where VZV is uncommon are likely to lack immunity either from a natural VZV infection or from childhood vaccinations. Some populations of native North Americans may also be at-risk, and include:

- Immune compromised/immunodeficient patients.
- Newborns of women who developed VZV infection shortly before (less than 5 days) or shortly after (within 2 days) delivery.
- Pregnant women.
- Premature infants.
- Full term infants less than 1 year of age.

The VZV infectivity and morbidity/mortality associated with VZV infections in these patient populations varies substantially (see **Table 1**).

Table 1: Incidence and Severity of VZV Infections in At-Risk Individuals

	Children[a]	Adults[b]	Pregnant Women[c]	Immunocompromised/ Immunodeficient[d]	Newborns, Pre-term Infants[e]
**VZV Infection Rates**	>70%	>70%	70-89%	88%	>50%
**Average Pox Counts**	~100	~300	Unknown	Unknown	Unknown
**Pneumonia (%)**	<1%	14-50%	14-50%	19-32%	18%
**Mortality (%)**	<0.001%	<1%	Unknown	7-50%	30%

References: a[5,9-11]; b[5,9-13]; c[10,11,14-16]; d[5,10,17-20]; e[9-11]

VZIG™ (Massachusetts Public Health Biological Laboratories), the VZIG product that was available in the USA until recently, was licensed based on treatment of VZV infection in immunocompromised children compared to untreated historical controls (**Table 2**).

Table 2: Incidence and Severity of VZV Infection in Immunocompromised Children

	Untreated Historical Controls	VZIG Treated (N=81)
VZV infection rate	88%	45%
Percent with >100 pox	87%	37%
Incidence of Pneumonia	25%	6%
Incidence of Encephalitis	5%	0%
Mortality	7%	1%

*References: 10,20

Controlled clinical studies involving high-risk neonates (including neonates (≤1 year of age), pre-term infants, newborns whose mothers had VZV infection shortly before (<5 days) delivery, and newborns whose mothers had VZV infection shortly after (<2 days) delivery, and healthy adults have not been done, but it is expected that VZV infection in these populations will also be attenuated by VZIG[10]. Based on these recommendations, the licensed VZIG product is labeled for each of the at-risk patient populations.

Clinical trials previously conducted by Cangene Corporation (study VZ-006) provided data suggesting that the safety and efficacy of VariZIG™ are similar to that of commercially

available VZIG in pregnant women exposed to VZV. Briefly, VariZIG™ and VZIG™ were similarly effective in reducing the incidence of VZV infection in pregnant women exposed to VZV. VariZIG™ also reduced mortality in this population, compared to untreated historical controls. Safety data for VariZIG™ was also collected during two additional trials, and indicated that VariZIG™ was well tolerated by normal healthy subjects (study VZ-001), and geriatric patients with post-herpetic neuralgia (study VZ-003).

VZIG™ has been licensed in the USA since 1980 for the passive immunization of exposed, susceptible individuals to reduce the incidence/severity of VZV infection. However, since the current manufacturer of VZIG has stopped producing this product, there is a need for a new varicella zoster immune globulin in the USA.

2.3 Hypothesis

VariZIG™ is safe and well tolerated in at-risk patients exposed to VZV infection. These at-risk patient populations include: immune compromised pediatric and adult patients, neonates (< 1 year of age), pre-term infants, pregnant women, and newborns of mothers with VZV infection shortly before (<5 days) or after (<2 days) delivery.

3 STUDY DESIGN

3.1 Study Design

This is an open-label expanded access protocol to assess the safety and efficacy of VariZIG™ in the prevention or reduction of complications resulting from varicella infections in at-risk patients exposed to individuals with contagious varicella infections. VariZIG™ will be distributed by [redacted] under this expanded access protocol. After determination of the need for VariZIG™ by the physician, the physician will contact Cangene Corporation (or designate) for approval and shipment of VariZIG™. Approval for the release of VariZIG™ will include contact with authorized Cangene Corporation personnel (or designate), and completion of a brief questionnaire. After either Cangene Corporation (or designate) reviews and approves the request, the physician's and patient's information will be entered into a database and VariZIG™, the Investigator's Brochure (IB), the protocol, the informed consent form (ICF), and trial documentation will be shipped from [redacted] to the physician. [redacted] will notify Cangene Corporation (or designate) of the shipment of VariZIG™ and the physician's information. VariZIG™ will then be then administered to the patient. Cangene Corporation (or designate) will contact the investigator within 7 days of VariZIG™ shipment for follow up and to discuss data collection. The patient (or legal representative) must sign an ICF prior to conducting any study procedures. Cangene Corporation (or designate) will contact the investigator by phone at least 3 times in order to collect data.

3.2 Anticipated Centers

Major centers that would potentially enroll patients in this study will be pre-identified and made aware of the study protocol procedures. Other centers will be determined on an individual patient basis.

3.3 Sample Size

This expanded access protocol is intended to provide VariZIG™ to all patients determined by their physician to be in need of this treatment, and who give their informed consent, until licensing of VariZIG™. Thus, the number of patients to be enrolled under this expanded access protocol is unknown. However, a minimum of 16 consented patients will be used in the efficacy analysis (see Section 8.1 for further details).

4 ELIGIBILITY REQUIREMENTS

4.1 Inclusion Criteria

- Signed informed consent.
- Cangene Corporation (or designate) VariZIG™ release requirement.
- Any of the following at-risk patients exposed to varicella:
  - Immune compromised pediatric patients.
  - Immune compromised adult patients.
  - Neonates (less than 1 year of age).
  - Pre-term infants.
  - Pregnant women.
  - Newborns whose mothers had VZV infection shortly before delivery (<5 days).
  - Newborns whose mothers had VZV infection shortly after delivery (<2 days).

4.2 Exclusion Criteria

- Hypersensitivity to blood or blood products, including IV or IM human immunoglobulin preparations.
- Selective IgA deficiency.
- Evidence of varicella infection.
- Evidence of Zoster infection.

4.3 Excluded Concomitant Medications

There are no restrictions on the use of concomitant medications during a course of treatment with VariZIG™.

5 STUDY PROCEDURES

5.1 Randomization

Since this study is an open-label design, patients will not be randomized.

5.2 Baseline and Baseline Assessment

Visit 1 (Baseline/Day 0)

Visit 1 (Baseline/Day 0) assessments should be performed by the physician or other designated healthcare provider. A log of concomitant medications including transfusions, herbal preparations and non-prescription medications must be kept throughout the study.

After receiving the shipment of VariZIG™ from [redacted], and prior to administration of VariZIG™ (Baseline/Day 0), the following assessments should be made:

- Signed informed consent.
- Basic medical history, especially information related to varicella exposure, including duration, type, and time since exposure.
- Collection of the following pre-treatment lab data, if available:
  - Hematology assessments: hemoglobin, hematocrit, WBC, RBC indices and platelet count.
  - Blood Chemistry: total bilirubin, AST, ALT, alkaline phosphatase, lactate dehydrogenase, creatinine, BUN.
- Administration of VariZIG™.

5.3 Drug Administration

Dosing of VariZIG™ is based on body weight. The recommended dose is 125 IU/10 kg body weight IM, to a maximum of 625 IU. This dose is to be used for patients greater than 5 kg body weight. For patients less than 5 kg body weight, 62.5 IU (one-half vial of VariZIG™) should be administered IM. This equals approximately 0.6 mL per dose, which will minimize any adverse effects in neonates.

VariZIG™ administration should occur within 96 hours of exposure to VZV. Treatment may be of uncertain value after 96 hours, based on the previously licensed product. Additional doses or dose adjustments may be utilized as clinically required. VariZIG™ may also be administered IV.

5.4 Study Assessment

Study assessments should be performed by the investigator or other designated healthcare provider. A log of concomitant medications including transfusions, herbal preparations and non-prescription medications must be kept throughout the study.

Visit 2 (Day 1 to Day 4)

Patients should return for assessment of the following between Day 1 and Day 4 after administration of VariZIG™:

- Evaluation of varicella lesion(s) (if applicable): type, number, size, location on the body and percent of body area affected.
- Collection of the following data if available:
  - Hematology assessments: hemoglobin, hematocrit, WBC, RBC indices and platelet count.
  - Blood Chemistry: total bilirubin, AST, ALT, alkaline phosphatase, lactate dehydrogenase, creatinine, BUN.
- Adverse event and concomitant medication information.

Visit 3 (Day 7 to 20 (Approximate day of varicella rash development as applicable)):

A patient who develops a varicella infection (rash) should return as soon as possible for evaluation. If infection occurs, it is expected between 11 and 20 days after exposure to VZV. Patients should return for assessment of the following between Day 7 and Day 20 after administration of VariZIG™, as is applicable by development of varicella rash:

- Review of varicella lesion(s): type, number, size, location of the body and percent of body area affected.
- Collection of the following data if available:
  - Hematology assessments: hemoglobin, hematocrit, WBC, RBC indices and platelet count.
  - Blood Chemistry: total bilirubin, AST, ALT, alkaline phosphatase, lactate dehydrogenase, creatinine, BUN.
- Adverse event and concomitant medication information.
- A patient who does not develop varicella should return for the closeout visit.

Visit 4 (Day 28 to 42 (closeout)):

All patients should return for the following assessments between Day 28 and Day 42 after administration of VariZIG™:

- Patients should return for a closeout visit upon resolution of the varicella (chicken pox) rash, or on Day 42, whichever occurs first. If the rash is not resolved by Day 42, the patient will be followed for safety and assessment of the varicella (chicken pox) rash. The return visits will be at the discretion of the investigator.
- Review of varicella lesion(s) (if applicable): type, number, size, location of the body and percent of body area affected.
- Clinical review of varicella infection severity including development of pneumonia, encephalitis and >100 pox on chest/back.
- Collection of the following data if available:
  - Hematology assessments: hemoglobin, hematocrit, WBC, RBC indices and platelet count.
  - Blood Chemistry: total bilirubin, AST, ALT, alkaline phosphatase, lactate dehydrogenase, creatinine, BUN.

- Adverse event and concomitant medication information.

5.5 Laboratory Assessments

Laboratory assessments will collect the following data if available:

- Hematology assessments: hemoglobin, hematocrit, WBC, RBC indices and platelet count.
- Blood Chemistry: total bilirubin, AST, ALT, alkaline phosphatase, lactate dehydrogenase, creatinine, BUN.

6 SAFETY ASSESSMENTS

6.1 Adverse Event/Serious Adverse Event Definition

The occurrence of adverse events (AE) will be monitored throughout all phases of the study and will cover all participating patients.

Adverse events are to be elicited by the physician (or designate) asking the patient non-leading questions. The association of the AE to VariZIG™ is to be judged by the physician as definite, probable, possible, unlikely or conditional.

6.1.1 Definitions

<u>An Adverse Event (AE):</u> Any untoward medical occurrence in a patient or clinical investigation subject administered a pharmaceutical product and that does not necessarily have a causal relationship with this treatment. An adverse event (AE) can therefore be any unfavorable and unintended sign (including an abnormal laboratory finding), symptom, or disease temporally associated with the use of a medicinal (investigational) product, whether or not related to the medicinal (investigational) product.

<u>A Serious Adverse Event (SAE):</u> Any untoward medical occurrence that at any dose: results in death, is life-threatening, requires inpatient hospitalization or prolongation of existing hospitalization, results in persistent or significant disability/incapacity, is a congenital anomaly/birth defect.

NOTE: Important medical events which may not result in death, be life threatening, or require hospitalization may be considered a SAE when, based upon appropriate medical judgment, they may jeopardize the patient or subject and may require medical or surgical intervention to prevent one of the outcomes listed in this definition.

Unexpected Adverse Drug Reaction: An adverse reaction, the nature or severity of which is not consistent with the applicable product information (e.g. Investigators Brochure for an unapproved investigational product or package insert/summary of product characteristics for an approved product).

All adverse events, including those that are not of a serious nature and those that are expected, will be documented by the investigators (or designates) on the source documents and appropriately transcribed onto separate data forms provided for this purpose. All adverse events will be examined by the investigator for assessment of both severity and causality using the following criteria:

6.1.2 Assessment of Severity (Intensity)

Mild: awareness of a sign or symptom but patient can tolerate.

Moderate: discomfort enough to cause interference with normal daily activity.

Severe: resulting in an inability to do work or do usual daily activity.

6.1.3 Assessment of Causality (WHO Definitions)

Definite Association: An event that follows a reasonable temporal sequence in relation to the administration of the drug or in which the drug level has been established in body fluids or tissues, that follows a known response pattern to the suspected drug; and that is confirmed by improvement upon stopping the drug (de-challenge) and reappears on repeated exposure (re-challenge).

Probable Association: An event that follows a reasonable temporal sequence in relation to the administration of the drug, that follows a known response pattern to the suspected drug and that is confirmed by de-challenge; and that cannot be reasonably explained by the known characteristics of the patient's clinical state. Re-challenge information is not required to fulfill this definition.

Possible Association: A clinical event, including laboratory test abnormality, with a reasonable time sequence to administration of the drug, but which could also be explained by concurrent disease or other drugs or chemicals. Information on drug withdrawal may be lacking or unclear.

Unlikely Association: A clinical event, including laboratory test abnormality, with a temporal relationship to drug administration which makes a causal relationship improbable, and in which other drugs, chemicals or underlying disease provide plausible explanations.

Conditional Association: A clinical event, including laboratory test abnormality, reported as an adverse reaction, about which more data is essential for a proper assessment or the additional data are under examination.

6.1.4 Description of Known Adverse Event Profile for VariZIG™.

Reactions to VariZIG™ are rare and mild in intensity. In the intended patient population, the most frequent treatment related adverse events were pain at the injection site, headache and rash. Other less frequent adverse reactions were myalgia, rigors, fatigue, nausea and flushing. The adverse event profile of VariZIG™ is expected to be comparable to other commercially available varicella zoster immune globulin (human) and intravenous immune globulin (human) products. The most common expected adverse drug reactions are chills, fever, headaches, vomiting, allergic reactions, nausea, arthralgia and moderate low back pain[20-24].

As is the case with all immune globulin drugs, there is a remote chance of an anaphylactic/anaphylactoid reaction with VariZIG™ in individuals with hypersensitivity to blood products. In the event of an immediate hypersensitivity reaction (anaphylaxis) characterized by collapse, rapid pulse, wheezing, difficulty breathing, pallor, cyanosis, edema or generalized urticaria, epinephrine should be administered followed by hydrocortisone and/or antihistamine, if necessary.

Clinical Trial Adverse Drug Reactions

See the current VariZIG™ Investigator's Brochure for complete details.

6.2 Adverse Event Reporting

Occurrence of adverse events will be monitored throughout all phases of the study at all visits and will cover all participating patients. Study patients should be provided with a 24hr telephone number to contact study personnel in case of an untoward reaction. The investigator(s) should follow all adverse events to resolution.

6.3 Reporting of SAEs

The investigator (or designate) will report all serious adverse and/or unexpected events to the sponsor within 24 hours of occurrence by telephone (i.e. within 24 hours of the investigator's (or designate's) knowledge of occurrence). This will be followed by a fax of the SAE Form. A written SAE report by the investigator to the sponsor (including medical summary of the SAE) must follow within 3 days of the investigator's (or designate's) knowledge of occurrence of the SAE. Telephone reports should be made to the contact listed below.

[redacted] OR [redacted]

Manager, Pharmacovigilance
Cangene Corporation
26 Henlow Bay
Winnipeg, MB R3Y 1G4
Phone: [redacted]
Fax: [redacted]
Cell: [redacted]

Safety Surveillance Officer
Cangene Corporation
26 Henlow Bay
Winnipeg, MB R3Y 1G4
Phone: [redacted]
Fax: [redacted]
Cell: [redacted]

Phone: [redacted] (North America only)
Fax: [redacted] (North America only)

7 STUDY MEDICATION

7.1 Medication Shipment

VariZIG™ is stored at [redacted]. Upon the physician's request, VariZIG™ will be shipped to the physician at a temperature of 36 to 46°F (2 to 8°C). The physician or designate will be responsible for checking the number of vials and the condition of the vials received.

7.2 Packaging and Formulation

VariZIG™ is supplied as a kit containing one 6 mL glass vial, which contains greater than 125 IU of freeze-dried VariZIG™, fitted with butyl rubber stopper and an aluminum seal with a plastic flip-off cap. Each package also contains 8.5 mL of sterile diluent, for reconstitution of VariZIG™. Refer to Section 7.5 of this protocol and the Investigator's Brochure for further details.

The final product formulation includes addition of sodium chloride to yield 0.04 M, glycine to yield 0.1 M and polysorbate 80 to yield 0.01%. The accompanying sterile diluent contains 0.8% sodium chloride and 10 mM sodium phosphate. The reconstituted product contains no preservative.

7.3 Labeling

VariZIG™ vial labels will include:

- Name of drug
- Lot number
- Expiration date
- Recommended storage conditions
- Name and address of manufacturer

- Name and address of distributor

The following will also be included:

"Caution: New Drug – Limited by Federal Law to Investigational Use"

7.4 Storage Conditions

Store VariZIG™ at 36 to 46°F (2 to 8°C). Do not use after expiry date. **Do not freeze.**

7.5 Preparation

VariZIG™ should be brought to room temperature immediately prior to use. Following reconstitution, the product should be clear or slightly opalescent. Do not use product that appears cloudy or contains deposits.

VariZIG™ should be reconstituted only with the accompanying vial of Sterile Diluent. Use aseptic technique throughout. Reconstitute shortly before use. To reconstitute:

1. Remove caps from the diluent and product vials
2. Wipe exposed central portion of each rubber stopper with suitable disinfectant
3. Withdraw the contents of the diluent using a suitable syringe and needle. For IM administration, reconstitute using a volume of 1.25 mL sterile diluent. For IV administration, reconstitute using a volume of 2.5 mL sterile diluent. (Refer to Investigator's Brochure for further details).
4. Inject diluent slowly at an angle so that the liquid is directed onto the inside glass wall of the vial containing the freeze-dried pellet.
5. Wet pellet by gently tilting and inverting the vial. Avoid frothing. Gently swirl upright vial until dissolved (less than ten minutes). **Do not shake.**

Reconstituted product can be stored for up to 12 hours at 36 to 46°F (2 to 8°C) prior to use.

Table 3: Reconstitution of VariZIG™

Route of Administration	Vial Size	Volume of Diluent to be added to vial	Approximate Available Volume	Nominal Concentration per mL
Intravenous (IV)	6 mL	2.5 mL	2.4 mL	50 IU/mL
Intramuscular (IM)	6 mL	1.25 mL	1.2 mL	100 IU/mL

7.6 Drug Accountability

The product supplied under this protocol is to be used solely for the treatment of the patient for which it is being released by [redacted] or Cangene Corporation.

The physician is responsible for submitting to [redacted] the necessary documents acknowledging receipt of the product, date of administration, dosage used and the indication for which it was used.

A research pharmacist or a designated individual will maintain a current inventory and ongoing record of test material supplies using the Drug Accountability Form provided by Cangene Corporation. This inventory record will include:

- Protocol name.
- Product name and description.
- Physician's name and site location.
- Product lot number.
- Number of vials dispensed, date of dispensing and patient for whom product was dispensed.
- Product balance.
- Name of qualified individual dispensing product.

These records may be reviewed by representatives of Cangene Corporation or the FDA.

8 STATISTICAL ISSUES IN STUDY DESIGN AND EFFICACY ASSESSMENT

8.1 Sample Size

All eligible consented patients will be enrolled in this study. There is no upper or lower limit for the number of patients enrolled. For evaluation of the secondary endpoint, a minimum of 16 immune compromised patients enrolled into this study will be compared to untreated historical control data for the incidence of pneumonia in at-risk patients exposed to VZV (chicken pox) infection. This will allow 80% power to detect a 25% difference between patients treated with VariZIG™ and historical control values.

8.2 Study Endpoints

The endpoints of this study are outlined below.

8.2.1 Primary Endpoint

The primary endpoint of this study is the incidence of adverse events associated with VariZIG™.

8.2.2 Secondary Endpoints

Secondary endpoints include:

- Incidence of mortality.
- Incidence of pneumonia.
- Incidence of encephalitis.
- Percent of patients with pox count >100.
- Overall incidence of varicella infection.
- Clinical observations and laboratory assessments.
- Safety profile of VariZIG™.

8.3 Planned Method of Analyses

Adverse events will be coded and listed. The incidence, intensity and relationship of events to VariZIG™ will be evaluated through the use of frequency tables. Adverse events will be tabulated by body system and preferred term, both in terms of number of occurrences and in terms of number of patients affected. Serious adverse events will be listed and tabulated in a similar fashion.

As a secondary endpoint analysis, historical rates of developing pneumonia in untreated patients will be compared to the observed rates of pneumonia development in patients in at-risk groups treated with VariZIG™. The at-risk group includes immunocompromised/ immunodeficient patients (Table 4). The superiority comparison will be performed using Fisher's exact test for proportions with a probability of type I error set at 0.05.

Table 4: Historical Rate of Development of Pneumonia in At-Risk Patients Untreated and Treated with VZIG

Risk Group	Untreated (% Development of Pneumonia)[20]	Treated with VZIG (% Development of Pneumonia)[20]
Immunocompromised/ Immunodeficient	25%	6%

Descriptive statistics will be provided for the percent of patients with a pox count >100, for the incidence of mortality, pneumonia and encephalitis in at-risk patients, and for the overall incidence of infection.

Concomitant medications will be coded and classified by an appropriate drug dictionary. These categories will be tabulated and summarized. Concomitant medications will also be listed individually, in full.

Demographic and baseline characteristics will be summarized using appropriate summary statistics.

9 REGULATORY AND ETHICAL ISSUES

9.1 Informed Consent

The investigator (or his/her representative) will obtain a written informed consent from prospective study candidates before enrollment or the performance of any study procedures. The proper completion of consent forms will be monitored by sponsor personnel and maintained in the study site trial master file. While obtaining informed consent from these patients, the investigators (or designates) will inform the patient of the following:

- That the trial involves research.
- The purpose of the trial.
- The trial treatment.
- The trial procedures to be followed, including all invasive procedures.
- The patient's responsibilities.
- Those aspects of the trial that are experimental.
- The reasonably foreseeable risks or inconveniences to the patient and, when applicable, to an embryo, fetus, or nursing infant.
- The reasonably expected benefits.
- The alternative procedure(s) or course(s) of treatment that may be available to the patient, and their important potential benefits and risks.
- The compensation and/or treatment available to the patient in the event of trial-related injury.
- The anticipated expenses, if any, to the patient for participating in the trial.
- That the patient's participation in the trial is voluntary and that the patient may refuse to participate or withdraw from the trial, at any time, without penalty or loss of benefits to which the patient is otherwise entitled.
- That the monitor(s), the auditor(s), the IRB, and the regulatory authority (ies) will be granted direct access to the patient's original medical records for verification of clinical trial procedures and/or data, without violating the confidentiality of the patient, to the extent permitted by the applicable laws and regulations and that, by signing a written informed consent form, the patient or the patient's legally acceptable representative is authorizing such access.

- That record identifying the patient will be kept confidential and, to the extent permitted by the applicable laws and/or regulations will not be made publicly available. If the results of the trial are published, the patient's identity will remain confidential.
- That the patient or the patient's legally acceptable representative will be informed in a timely manner if information becomes available that may be relevant to the patient's willingness to continue participation in the trial.
- The person(s) to contact for further information regarding the trial and the rights of trial patients, and whom to contact in the event of trial-related injury.
- The foreseeable circumstances and/or reasons under which the patient's participation in the trial may be terminated.
- The expected duration of the patient's participation in the trial.
- The approximate number of patients involved in the trial.

9.2 Institutional Review Board (IRB)

Before the start of the study, investigator's brochure, the protocol, and proposed informed consent forms will be submitted to a properly constituted Institutional Review Board (IRB) for written approval. Cangene Corporation (or designate) must receive a copy of the written approval from the Central IRB for the protocol, investigator's brochure and informed consent form prior to recruitment of patients into the study.

The Central IRB must provide written approval for all amendments to the protocol and the written informed consent form prior to implementation of these amendments at the investigational site.

The names and associated backgrounds (to assist in assuring the board membership complies with ICH requirements) of the members of the Central IRB will be given to the sponsor (Cangene Corporation) prior to the start of the trial along with a signed and dated statement stating that the protocol and Informed Consent Form have been approved by them. This board or committee will review and approve all amendments to the protocol. Where applicable, the investigator will notify their local IRB, provide a description of the local IRB requirements, and provide documentation of local IRB approval to Cangene Corporation (or designate).

All correspondence between the investigator and the IRB will be available for review by Cangene Corporation (or designate).

9.3 Study Documentation

The following documentation will be provided to the investigator with the shipment of VariZIG™:

- Protocol
- Investigator's Brochure
- FDA 1572 form
- Financial Disclosure Form
- Copy of approved informed consent form.
- Copy of the central IRB approval of protocol, investigator brochure, informed consent form and any recruiting material.
- Flow diagram and contact information.
- Description of how to enter patient information into electronic data forms (see section 11.5).

The following documentation should be completed and returned to Cangene Corporation as soon as possible following receipt of the VariZIG™ shipment:

- Signed FDA 1572 form
- Signed protocol
- Signed Financial Disclosure Form
- Investigator's (and sub-investigator's) curriculum vitae (signed and dated), copy of medical license
- Entry of data into electronic data capture system

9.4 Patient Confidentiality

The investigator must ensure the anonymity of each patient is maintained at all times. Patients should only be identified by their initials and randomization number on the CRF. Any documents that identify the patient should be kept in strict confidence by the principal investigator.
Based on ICH GCP guidelines and regulatory requirements, the investigator is required to allow authorized personnel of Cangene Corporation or its designate and of the appropriate regulatory authority (ies) to review patient's files that are related to study VZ-009. Patients must be informed that his/her records may be reviewed by Cangene Corporation, its designate and the appropriate regulatory authority.

10 ADMINISTRATIVE AND LEGAL REQUIREMENTS

10.1 Sponsorship

This clinical study is sponsored by the manufacturer (Cangene Corporation, Winnipeg) of VariZIG™.

10.2 Protocol Amendments

Protocol amendments will only be made by Cangene Corporation. The central IRB must be informed of all protocol amendments and provide approval for any amendments that may affect the safety of patients or the conduct of the study.

10.3 Study Termination

Cangene Corporation may terminate the study at its discretion. Cangene Corporation must notify the central IRB in writing of the trial's completion or early termination.

10.4 Source Documentation and Storage

The principal investigator will maintain the following information:

- Medical history/physical condition of the study patient before involvement in the study sufficient to verify protocol entry criteria.
- Dated and signed notes on the day of entry into the study including the study number, the drug being evaluated, patient number assigned, and a statement that informed consent was obtained, noting the time the consent was obtained.
- Dated and signed notes from each study patient visit referring to the protocol and CRFs for further information, if appropriate (for specific results of procedures and exams).
- Notes regarding concomitant medications taken during the study (including start and stop dates).
- Patients' condition upon completion, or withdrawal from, the study.
- All communications with the IRB responsible for the study.

Records from the study that identify the patient will be confidential except that they may be given to and inspected by the sponsor of the study, the Food and Drug Administration, other government agencies, and will not otherwise be released except as required by law. All information provided to the investigator by the sponsor is to be considered confidential unless otherwise stated.

10.5 Electronic Data Capture

A web based electronic data capture system will be used for collecting patient information, including demographics, type of exposure to VZV, safety information, and varicella-specific information (such as assessment of the varicella rash, including type, number, size, location on the body, and percent of the body area affected). Instructions for entry of data into the electronic data capture system will be included with the VariZIG™ shipment

10.6 Publication

Data arising from this study is the sole property of the sponsor of the study, Cangene Corporation. The sponsor must provide written, prior agreement to any publication based, in whole or in part, on data from this study.

11 REFERENCES

1. Bowman JM, Friesen, AS, Pollock JM, Taylor WE. WhinRho: RH immune globulin prepared by ion exchange for intravenous use. Can Med Assoc J 1980; 123(11):1121-1127.

2. Friesen A, Bowman J, Price H. Column ion-exchange preparation and characterization of an Rh immune globulin (WinRho) for intravenous use. J Appl Biochem 1981; 3(2):164-175.

3. Whitley RJ. Therapeutic approaches to varicella-zoster virus infections. J Infect Dis 1992; 166 Suppl 1:S51-S57.

4. Ross A. Modification Of Chicken Pox In Family Contacts By Administration Of Gamma Globulin. New Engl J Med 1962; 267(8):369-376.

5. Hockberger RS, Rothstein RJ. Varicella pneumonia in adults: a spectrum of the disease. Ann Emerg Med 1986; 15(8):93-934.

6. Orenstein WA, Heymann DL, Ellis RJ, Rosenberg RL, Nakano J, Halsey NA et al. Prophylaxis of varicella in high-risk children: dose-response effect of zoster immune globulin. J Pediatr 1981; 98(3):368-373.

7. Wallace MR, Bowler WA, Murray NB, Brodine SK, Oldfield EC, III. Treatment of adult varicella with oral acyclovir. A randomized, placebo-controlled trial. Ann Intern Med 1992; 117(5):358-363.

8. Gershon AA, Raker R, Steinberg S, Topf-Olstein B, Drusin LM. Antibody to Varicella-Zoster virus in parturient women and their offspring during the first year of life. Pediatrics 1976; 58(5):692-696.

9. Meyers JD. Congenital varicella in term infants: risk reconsidered. J Infect Dis 1974; 129(2):215-217.

10. Varicella-zoster immune globulin for the prevention of chickenpox. Recommendations of the Immunization Practices Advisory Committee, Centers for Disease Control. Ann Intern Med 1984; 100(6):859-865.

11. Preblud SR. Age-specific risks of varicella complications. Pediatrics 1981; 68(1):14-17.

12. Triebwasser JH, Harris RE, Bryant RE, Rhoades ER. Varicella pneumonia in adults. Report of seven cases and a review of literature. Medicine (Baltimore) 1967; 46(5):409-423.

13. Weber D, Pellecchia J. Varicella Pneumonia. JAMA 1965; 192(6):572-573.

14. Harris R, Rhoades E. Varicella Pneumonia Complicating Pregnancy. Obstetrics and Gynecology 1964;734-740.

15. Paryani SG, Arvin AM. Intrauterine infection with varicella-zoster virus after maternal varicella. N Engl J Med 1986; 314(24):1542-1546.

16. Russell LK. Management of varicella-zoster virus infection during pregnancy and the peripartum. J Nurse Midwifery 1992; 37(1):17-24.

17. Perronne C, Lazanas M, Leport C, Simon F, Salmon D, Dallot A et al. Varicella in patients infected with the human immunodeficiency virus. Arch Dermatol 1990; 126(8):1033-1036.

18. Straus SE, Ostrove JM, Inchauspe G, Felser JM, Freifeld A, Croen KD et al. NIH conference. Varicella-zoster virus infections. Biology, natural history, treatment, and prevention. Ann Intern Med 1988; 108(2):221-237.

19. Balfour HH Jr. Varicella zoster infections in immunocompromised hosts. A review of the natural history and management. Am J Med 1988; 85(2A):147(4):737-743.

20. Zaia JA, Levin MJ, Preblud SR, Leszczynski J, Wright GG, Ellis RJ et al. Evaluation of varicella-zoster immune globulin: protection of immunosuppressed children after household exposure to varicella. J Infect Dis 1983; 147(4):737-743.

21. Roche B, Samuel D. Liver transplantation for hepatitis B virus-related liver disease: indications, prevention of recurrence and results. J Hepatol 2003; 39 Suppl 1:S181-S189.

22. Hong F, Ruiz R, Price H, Griffiths A, Malinoski F, Woloski M. Safety profile of WinRho anti-D. Semin Hematol 1998; 35(1 Suppl 1):9-13.

23. Bussel JB, Eldor A, Kelton JG, Varon D, Brenner B, Gillis S et al. IGIV-C, a novel intravenous immunoglobulin: evaluation of safety, efficacy, mechanisms of action, and impact on quality of life. Thromb Haemost 2004; 91(4):771-778.

24. Scaradavou A, Woo B, Woloski BM, Cunningham-Rundles S, Ettinger LJ, Aledort LM et al. Intravenous anti-D treatment of immune thrombocytopenic purpura: experience in 272 patients. Blood 1997; 89(8):2689-2700.
